# Supplementary material for: High-Chern-number and high-temperature quantum Hall effect without Landau levels
Source: Natl Sci Rev. 2020 Apr 30;7(8):1280–7. doi: 10.1093/nsr/nwaa089 (PMC8289033; doi:10.1093/nsr/nwaa089)
Supplement: nwaa089_Supplemental_File [file nwaa089_supplemental_file.docx]

Supplementary Information for

High-Chern-Number and High-Temperature Quantum Hall Effect without Landau Levels

Jun Ge, Yanzhao Liu, Jiaheng Li, Hao Li, Tianchuang Luo, Yang Wu, Yong Xu, Jian Wang

Correspondence to: jianwangphysics@pku.edu.cn (J.W.); yongxu@mail.tsinghua.edu.cn (Y.X.)

**This PDF file includes:**

Supplementary Theoretical Discussions

Figs. S1 to S10

References (1, 2)

**Supplementary Theoretical Discussions**

**The occurrence of high-Chern-number in ferromagnetic MnBi_2_Te_4_ thin films**

Theoretically, a discrete increase of Chern number with increasing film thickness is a generic feature of ferromagnetic Weyl semimetal. As far as we know, there exists only one another topological phase, the 3D quantum anomalous Hall (QAH) insulator that can also give such kind of topological feature. However, our first-principles calculations prove that the 3D QAH insulator is unlikely to appear in the ferromagnetic MnBi_2_Te_4_. Moreover, for 2D films from a 3D QAH insulator, each unit layer would contribute a nonzero quantized Chern number, which is clearly not the case in our experiment. Therefore, our combined theoretical and experimental results strongly indicate that the ferromagnetic MnBi_2_Te_4_ is a magnetic Weyl semimetal.

In the following part, we present two physical pictures to understand the thickness-dependent Chern number in thin films of ferromagnetic MnBi_2_Te_4_:

1. **Topological band inversion picture:**

It is well known that for 2D films of a 3D topological insulator, the band gap as a function of film thickness displays an oscillating change behavior due to the existence of band inversion in the bulk, and each band gap closing-reopening corresponds to a topological phase transition, leading to an oscillating variation of the topological *Z_2_* invariant [1]. The same argument is applicable to a 3D ferromagnetic Weyl semimetal that also has an inverted band structure [2]. Differently, topology of 2D films from 3D ferromagnetic Weyl semimetal is described by Chern number, which is a topological *Z* invariant and thus can increase discretely with film thickness.

To explicitly demonstrate the physical picture, we performed theoretical calculations on MnBi_2_Te_4_ films by the effective Hamiltonian method. For simplicity, we focused on the center of the surface Brillouin zone (i.e., *k_x_=k_y_*=0), where the band inversion of 2D films happens. We first derived an effective two-band Hamiltonian to describe the low energy physics of the ferromagnetic Weyl semimetal state along the Γ-Z direction:

$H(k_{z})=\left[\begin{matrix} M_{1}+t_{1}k_{z}^{2} & 0 \\ 0 & M_{2}+t_{2}k_{z}^{2} \end{matrix}\right]$.

The parameters fitted from the *ab initio* band structure are

$M_{1}=-0.004\text{eV, }M_{2}=0.047\text{eV},\text{ }t_{1}=2.74\text{eV}\cdot\text{Å}^{2},\text{ }t_{2}=-15.10\text{eV}\cdot\text{Å}^{2}$.

Following [1], we calculated the energy levels versus the thickness of films by the effective Hamiltonian method. As displayed in Fig. S10a, the band gap regularly closes and reopens when increasing the film thickness, resulting in topological quantum phase transitions. Consequently, the anomalous Hall conductance (or Chern number) of 2D films indeed increases discretely with film thickness as shown in Fig. S10b. Note that the numerical results from the effective Hamiltonian method and the *ab initio* calculations have minor differences, presumably because the former approach cannot properly describe 2D films in the ultrathin limit.

2) **Anomalous Hall conductance picture**:

For the bulk of ferromagnetic MnBi_2_Te_4_, the averaged anomalous Hall conductance per unit layer $\bar{\sigma}_{xy}$ has a finite value (see the main text). $\sigma_{xy}$ of 2D films thus grows with the film thickness. On the other hand, $\sigma_{xy}$ of 2D gapped films must take quantized values as topologically required. This enforces a discrete increase of Chern number with increasing film thickness.


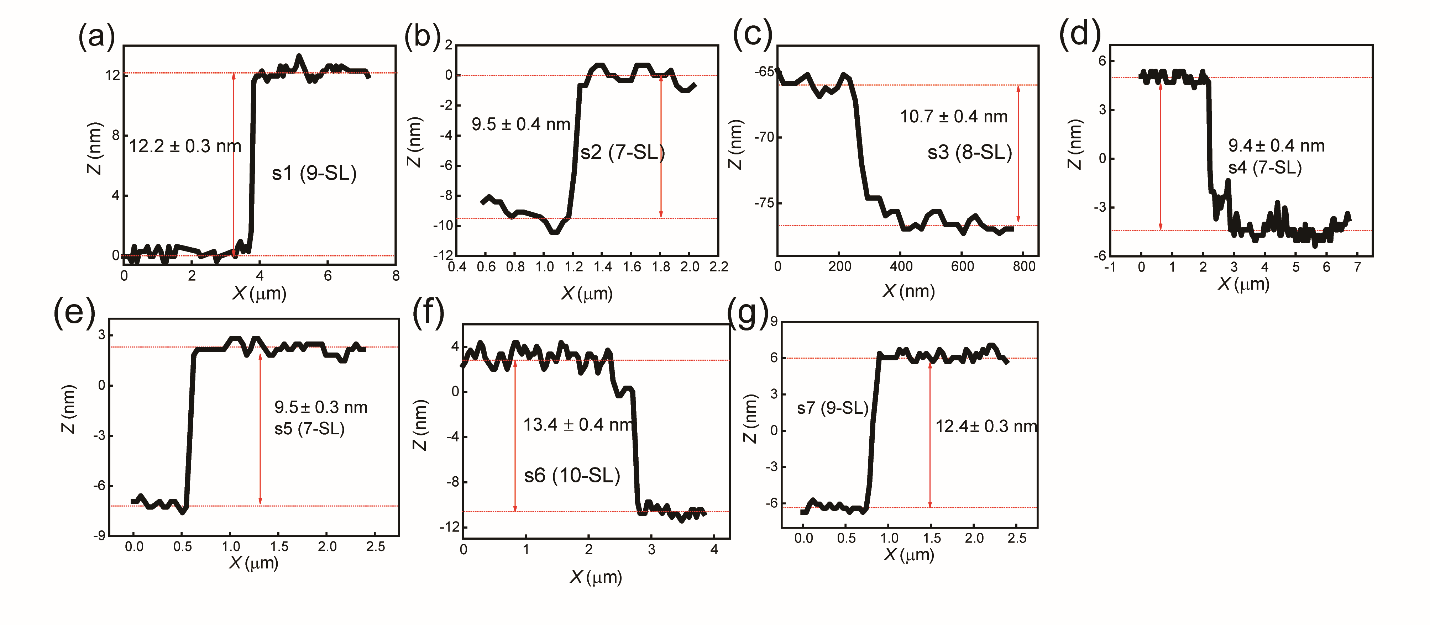


**Figure S1.** Thickness of MnBi_2_Te_4_ devices s1-s7, where high-Chern-number and high-temperature Chern insulator states are detected, measured by atomic force microscope (AFM).


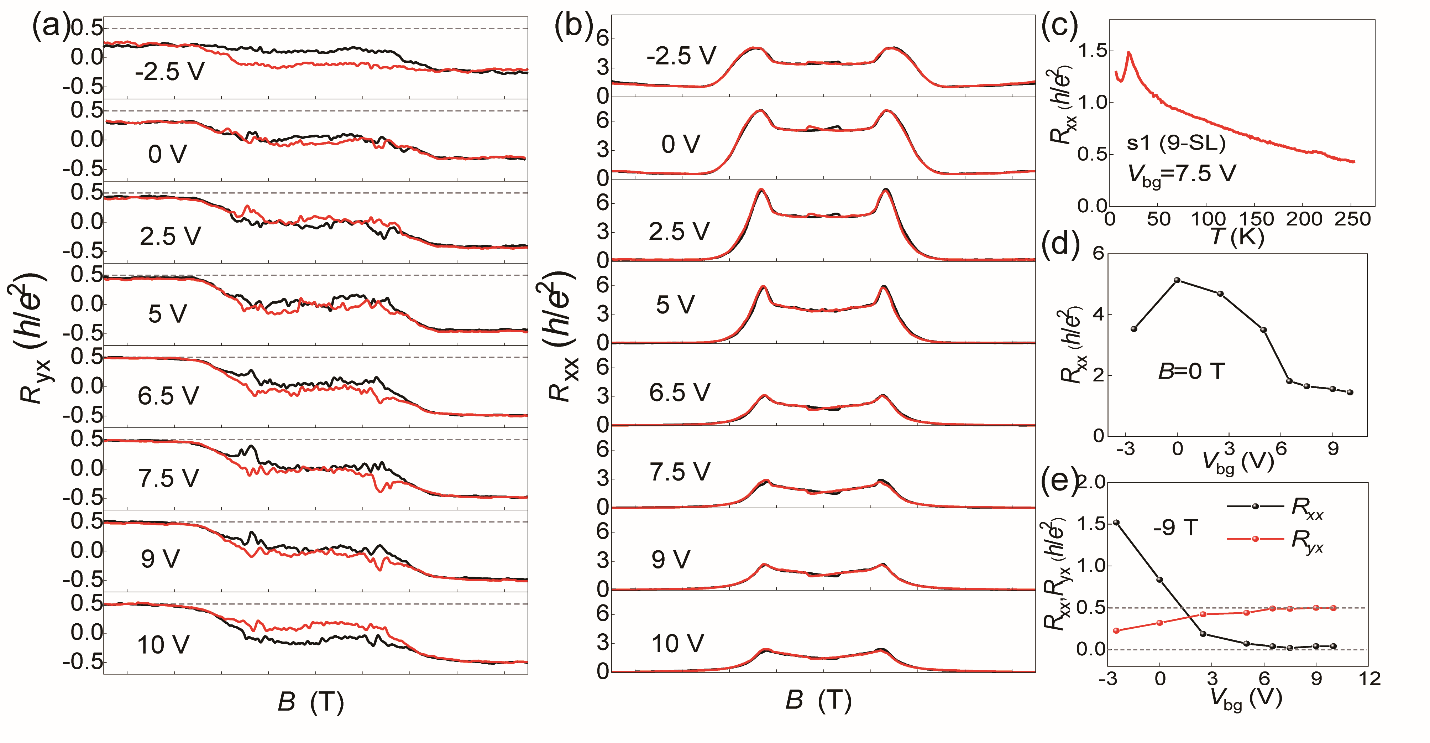


**Figure S2.** High-Chern-number QHE without LLs in MnBi_2_Te_4_ device s1 (9-SL). (a, b) *R*_yx_ and *R*_xx_ as a function of magnetic field at different back gate voltages *V*_bg_ at 1.9 K. (c) Temperature dependence of *R*_xx_ at *V*_bg_ =7.5 V. *T*_N_ is identified as 20 K. (d) *R*_xx_ vs. *V*_bg_ at zero magnetic field. (e) *R*_yx_ and *R*_xx_ as a function of *V*_bg_ at 1.9 K and -9 T.


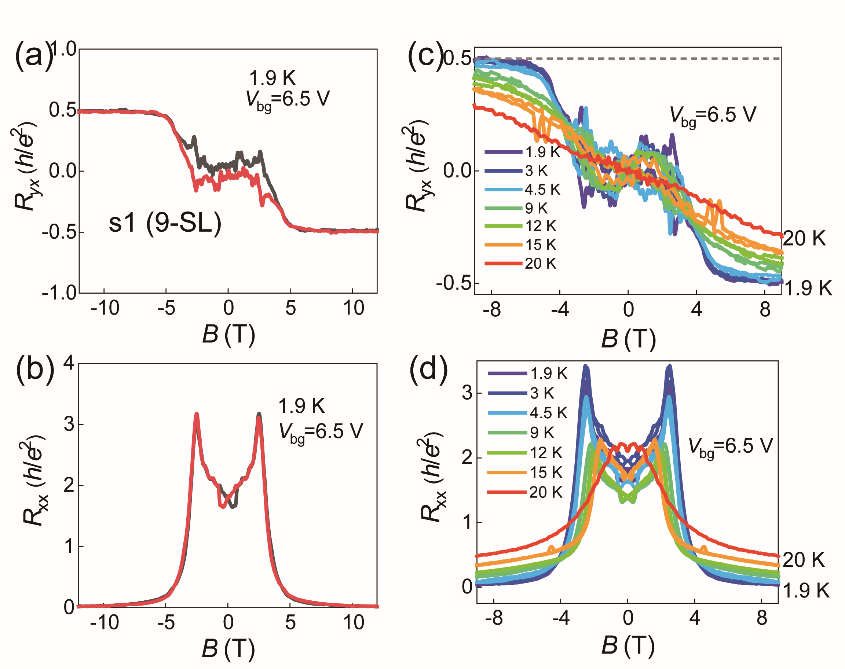


**Figure S3**. Temperature dependence of the high-Chern-number QHE without LLs in s1 (9-SL). (a, b) High-Chern-number QHE without LLs under the magnetic field up to 12 T at 1.9 K. (c, d) *R*_yx_ and *R*_xx_ as a function of magnetic field at different temperatures from 1.9 K to 20 K. From bottom: *T*=1.9 K, 3.0 K, 4.5 K, 9.0 K, 12 K, 15 K, 20 K.


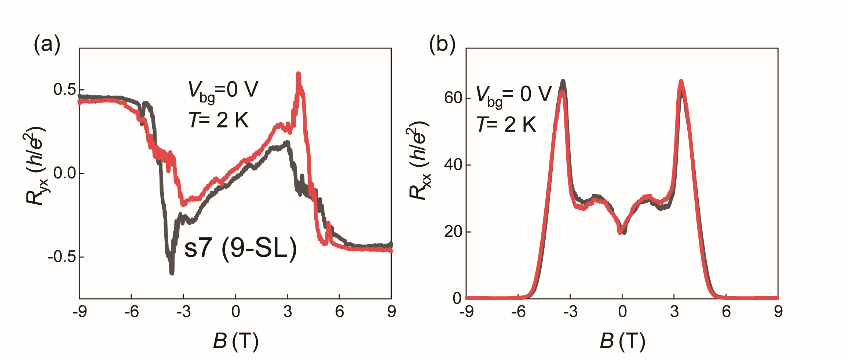


**Figure S4.** High-Chern-number QHE without LLs in MnBi_2_Te_4_ device s7 (9-SL). (a, b) *R*_yx_ and *R*_xx_ as a function of magnetic field at 2 K, 0 V. The nearly quantized Hall resistance plateau with height of 0.92 *h*/*2e*^2^ and *R_xx_* of 0.42 *h*/*2e*^2^ are detected.


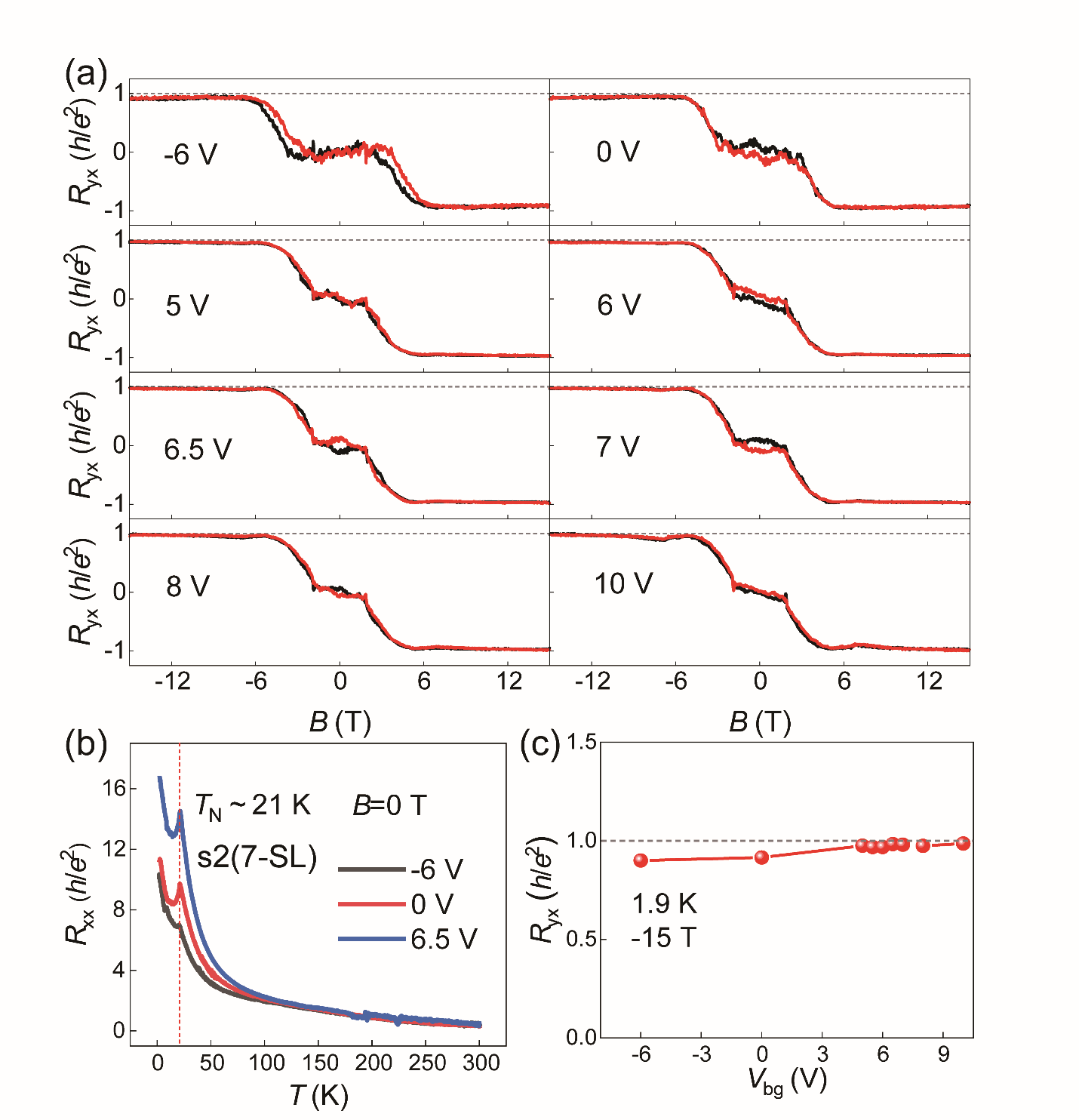


**Figure S5.** Gate-dependent transport properties of s2 (7-SL) with *C*=1. (a) The gate-dependent evolution of the *C*=1 QHE without LLs measured at 1.9 K. (b) *R*_xx_ as a function of temperature at different back gate voltages *V*_bg_ without magnetic field in s2. *T*_N_ is identified as 21 K. (c) *R*_yx_ as a function of *V*_bg_ at 1.9 K, -15 T.


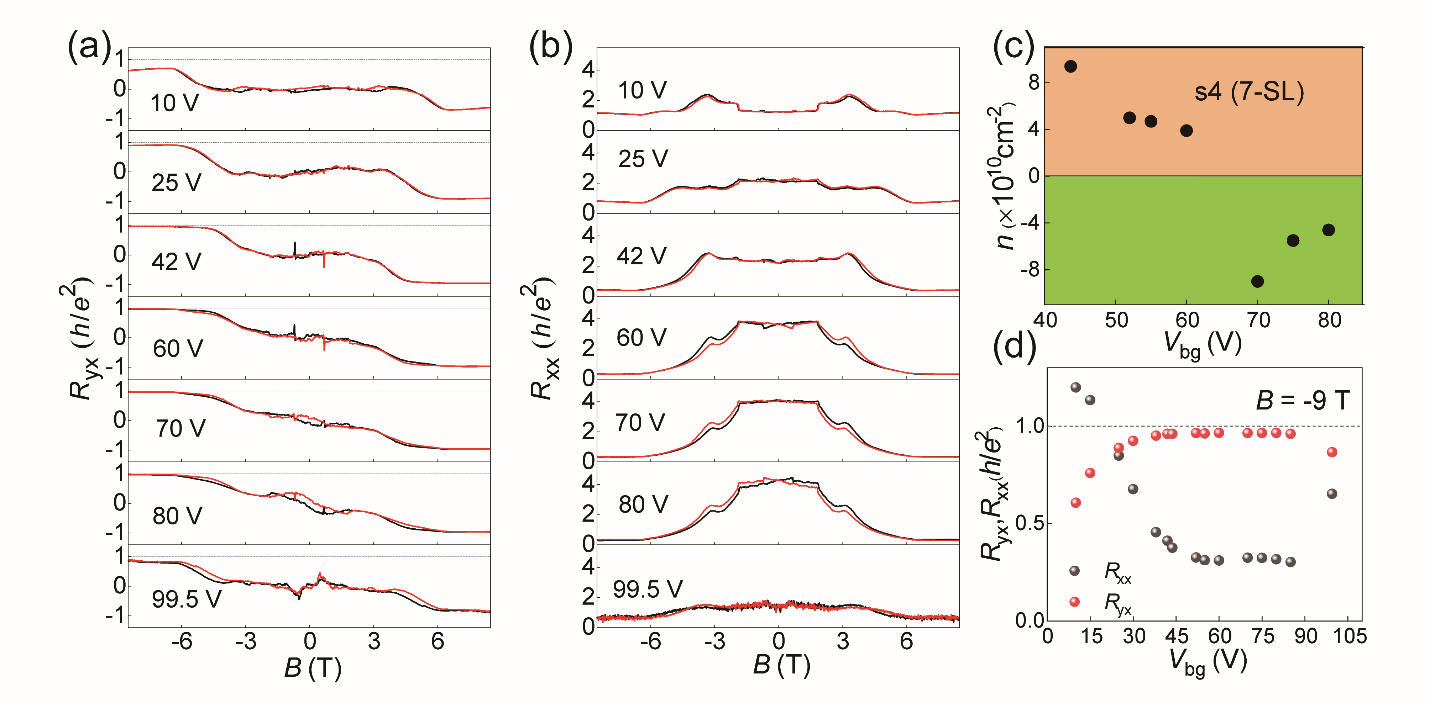


**Figure S6. QHE without LLs in MnBi_2_Te_4_ device s4 (7-SL) with *C*=1.** (a, b) *R*_yx_ and *R*_xx_ as a function of magnetic field at selected back gate voltages *V*_bg_ at 2 K. Quantized Hall resistance plateau and nearly vanishing *R*_xx_ are detected. (c) Carrier density *n* plotted as a function of *V*_bg_. *n* is estimated from fitting *R*_yx_ near zero magnetic field. (d) *R*_yx_ and *R*_xx_ as a function of *V*_bg_ at 2 K and -9 T. The quantized Hall resistance is limited within 40 V-85 V, which further confirms that the observed quantized Hall resistance plateau is indeed Chern insulator state. The carrier type is tuned from *p* to *n* when *V*_bg_ is modulated from 60 V to 70 V, while the sign of quantized *R*_yx_ plateau does not change.


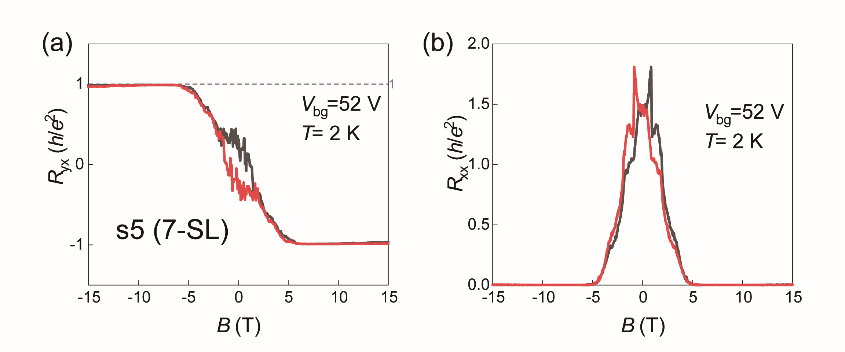


**Figure S7.** QHE without LLs in s5 (7-SL) with *C*=1. (a, b) *R*_yx_ and *R*_xx_ as a function of magnetic field at 2 K, 52 V. The quantized Hall resistance plateau with height of 0.984 *h*/*e*^2^ and *R*_xx_ of 0.003 *h*/*e*^2^ are detected.


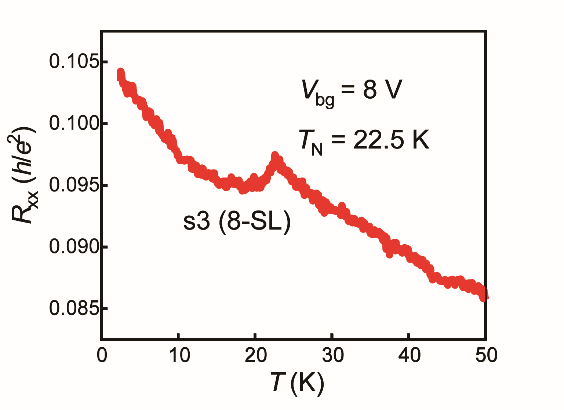


**Figure S8.** *R*_xx_ in s3 (8-SL) as a function of temperature from 50 K to 1.9 K at *V*_bg_ =8 V. *T*_N_ is identified as 22.5 K.


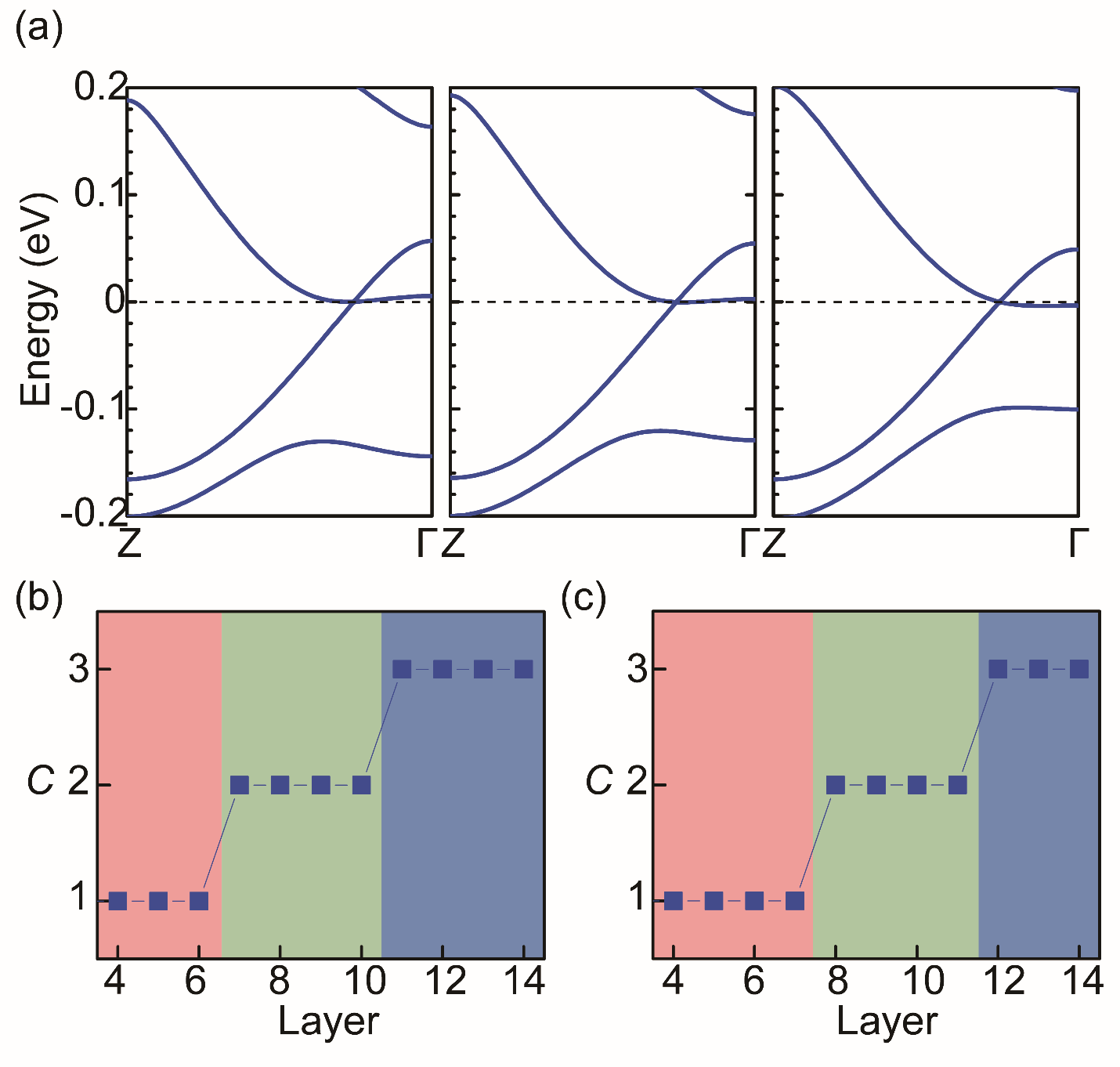


**Figure S9.** Band structure of bulk and topological Chern number of thin films for ferromagnetic MnBi_2_Te_4_ with varying out-of-plane lattice constant *c =* 3*c*_0_. Lattice constants obtained from first-principles calculations are *a* = 4.36$Å$ and *c*_0_ = 13.53$Å$. *c*_0_ is manually varied from the theoretical value to the experimental value (*c*_0_ = 13.6$Å$). (a) Band structures of bulk with *c*_0_ = 13.53$Å$ (left panel), *c*_0_ = 13.55$Å$ (middle panel) and *c*_0_ = 13.60$Å$ (right panel). (b, c) Topological Chern number as a function of film thickness for *c*_0_ = 13.53$Å$ and *c*_0_ = 13.55$Å$, respectively.


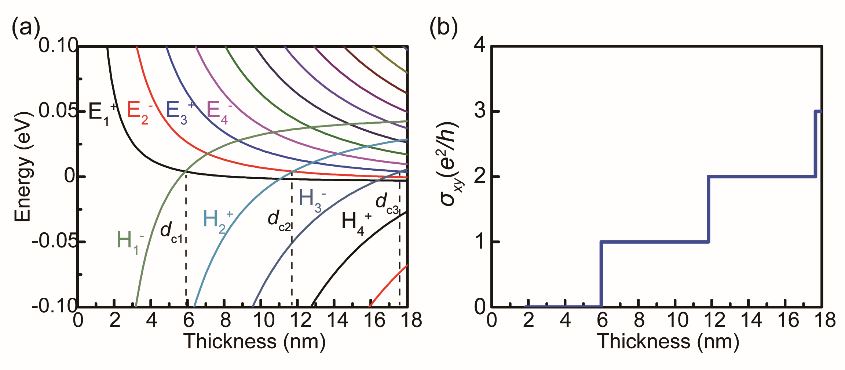


**Figure S10.** (a) The energy level at Γ versus the thickness of MnBi_2_Te_4_ films and (b) the anomalous Hall conductance as a function of film thickness calculated by the effective Hamiltonian method.

References

1. Liu, CX. Zhang H and Yan B *et al*. Oscillatory crossover from two dimensional to three dimensional topological insulators. *Phys Rev B* 2010; **81**: 041307-10 (R).
2. Xu G, Weng H and Wang Z *et al*. Chern semimetal and the quantized anomalous Hall effect in HgCr_2_Se_4_. *Phys Rev Lett* 2011;**107**: 186806-10.
